# THE ROLE OF HEALTH CARE STEREOTYPE THREAT IN END-OF-LIFE PLANNING AMONG OLDER SEXUAL MINORITY ADULTS

**DOI:** 10.1093/geroni/igae098.0754

**Published:** 2024-12-31

**Authors:** Meki Singleton

**Affiliations:** Rutgers University, East Orange, New Jersey, United States

## Abstract

Healthcare stereotype threat (HCST), defined as “the threat of being reduced to group stereotypes within healthcare encounters”, may occur when social identities negatively impact healthcare experiences. Prior research has shown that individuals report experiencing HCST related to age, gender, weight, race/ethnicity, HIV status, and sexual orientation. However, little is known about how HCST may be associated with end-of-life care planning. The aim of this study was to examine the association between comfort with medical decision making and advance care planning (ACP) activities and HCST related to sexual orientation and race. We conducted an online survey from 2022—2023 to collect information about older sexual minority (SM) adults’ ACP activities and perceptions of HCST. Respondents (N=281) included Black (n=133) and White (n=148) SM adults. Logistic regression revealed that those with higher levels of HCST–sexual orientation had 13% lower odds of being very comfortable with medical decision-making (p< 0.001) and 6% higher odds of having ACP discussions (p< 0.05). Higher rates of HCST–race was associated with 8% lower odds of being very comfortable with medical decision-making (p< 0.05) and 10% higher odds of having ACP discussions (p< 0.001), 7% higher odds of having completed a living will (p< 0.05), and 10% higher odds of having designated a healthcare proxy (p< 0.001). Findings demonstrate that HCST may negatively impact comfort in healthcare decision-making while also potentially motivating older SM adults to formally engage in ACP. Research is needed to investigate the barriers and challenges to engaging in ACP among older SM adults and interventions to reduce HCST.

